# Combining Anti-Mitochondrial Antibodies, Anti-Histone, and PLA2/COX Biomarkers to Increase Their Diagnostic Accuracy for Autism Spectrum Disorders

**DOI:** 10.3390/brainsci14060576

**Published:** 2024-06-05

**Authors:** Afaf El-Ansary, Hanan A. Alfawaz, Abir Ben Bacha, Laila Y. Al-Ayadhi

**Affiliations:** 1 Autism Center, Lotus Holistic Alternative Medical Center, Abu Dhabi P.O. Box 110281, United Arab Emirates; 2 Department of Food Science and Nutrition, College of Food & Agriculture Sciences, King Saud University, P.O. Box 22452, Riyadh 11495, Saudi Arabia; halfawaz@ksu.edu.sa; 3 Department of Biochemistry, College of Science, King Saud University, P.O. Box 22452, Riyadh 11495, Saudi Arabia; aalghanouchi@ksu.edu.sa; 4 Department of Physiology, Faculty of Medicine, King Saud University, P.O. Box 2925, Riyadh 11461, Saudi Arabia

**Keywords:** autism spectrum disorder, phospholipase A2, cyclooxygenase-2, anti-mitochondrial antibodies, anti-histone autoantibodies, mitochondrial dysfunction, glutamate excitotoxicity

## Abstract

Background: Autism spectrum disorder (ASD) is a neurodevelopmental disorder characterized by impairments in social interaction and restricted and repetitive behaviors. Oxidative stress may be a critical link between mitochondrial dysfunction and ASD as reactive oxygen species (ROS) generated from pro-oxidant environmental toxicants and activated immune cells can result in mitochondrial failure. Recently, mitochondrial dysfunction, autoimmunity, and abnormal lipid mediators have been identified in multiple investigations as an acknowledged etiological mechanism of ASD that can be targeted for therapeutic intervention. Methods: The relationship between lipid mediator markers linked to inflammation induction, such as phospholipase A2/cyclooxygenase-2 (PLA2/Cox-2), and the mitochondrial dysfunction marker anti-mitochondrial antibodies (AMA-M2), and anti-histone autoantibodies in the etiology of ASD was investigated in this study using combined receiver operating characteristic (ROC) curve analyses. This study also sought to identify the linear combination for a given set of markers that optimizes the partial area under ROC curves. This study included 40 age- and sex-matched controls and 40 ASD youngsters. The plasma of both groups was tested for PLA2/COX-2, AMA-M2, and anti-histone autoantibodies’ levels using ELISA kits. ROC curves and logistic regression models were used in the statistical analysis. Results: Using the integrated ROC curve analysis, a notable rise in the area under the curve was noticed. Additionally, the combined markers had markedly improved specificity and sensitivity. Conclusions: The current study suggested that measuring the predictive value of selected biomarkers related to mitochondrial dysfunction, autoimmunity, and lipid metabolism in children with ASD using a ROC curve analysis could lead to a better understanding of the etiological mechanism of ASD as well as its relationship with metabolism.

## 1. Introduction

Autism spectrum disorders (ASDs) are neurodevelopmental diseases characterized by varied degrees of defective communication and social abilities, repetitive and stereotypical behaviors, and attention, cognitive, learning, and sensory deficiencies. The most recent prevalence of autism spectrum disorder is 1 in 36, which is dramatically increased above earlier estimates [1].

The defining feature of autoimmune disorders is autoantibodies, which are essential to the initial stages of the diagnostic process. It is interesting to note that autoantibodies can exist for years prior to the onset of symptoms [2]. Targeting lipoic acid-containing immunodominant epitopes, anti-mitochondrial antibodies (AMAs) specifically target the E2 subunits of 2-oxo acid dehydrogenase complexes. However, there are a number of antigens in the inner mitochondrial membrane that AMA may target; however, their precise clinical and diagnostic importance is yet unknown [3]. AMA can be linked to mitochondrial dysfunction as a well-known etiological mechanism of ASD consequent to oxidative stress [4]. The overproduction of reactive oxygen species during oxidative stress has been shown to produce mitochondrial DNA mutations, disrupt the mitochondrial respiratory chain, alter membrane permeability, and impair Ca^2+^ homeostasis and mitochondrial defense mechanisms.

Histones, important proteins, act as a wrapper around DNA, controlling gene expression [5,6]. Post-translational modifications to histone proteins alter their interaction with DNA and other nuclear proteins, causing self-immunity [7]. Although the functional role of anti-histone autoantibodies is uncertain, they may have proteolytic activity on histone proteins [8]. Accordingly, it is unclear if this has a direct impact on disease pathogenesis [8]. Mecocci et al. [9] hypothesized that the presence of anti-histone antibodies in dementias could indicate a shift in membrane fluidity and integrity caused by nuclear immunogen outflow or immune response abnormalities. In the case of a disrupted blood–brain barrier in autistic patients, the influx of anti-histone antibodies from blood to the brain and their binding to membrane phospholipids could lead to neuronal damage either by the direct interaction of these complexes with neurons or by functional impairment due to their interaction with astrocytes, activation of endothelial cells, and adherence to different brain cells. Histones bind firmly to anionic phospholipids and are quickly released upon cell injury [10]. D’Angelo et al. [11] imply that autoantibodies against histones or histone–phospholipid complexes could contribute to anti-phospholipid activity.

Phospholipase A2s (PLA2s) are lipolytic enzymes that hydrolyze the acyl bond at the sn-2 position of glycerophospholipids, producing free fatty acids and lysophospholipids. The brain and spinal cord contain many PLA2 isoforms with varying activity, demonstrating PLA2’s significance in the physiology of the central nervous system (CNS). High levels of PLA2 and its reaction products are associated with altered membrane permeability, lipid peroxide accumulation, oxidative stress, and neuronal death [12], despite PLA2’s importance in physiological processes like phospholipid turnover, membrane remodeling, neurotransmitter release, and signal transduction [12].

Cyclooxygenase (COX) enzymes catalyze the conversion of arachidonic acids (AAs) into prostanoids. COX-1 is constitutively expressed by neurons, astrocytes, and microglia in the CNS, whereas COX-2 is expressed by glutamatergic neurons in the cerebral cortex, hippocampus, and amygdala and can be induced in other cell types [13,14]. COX-2 and its derivatives serve key physiological roles in synaptic plasticity and long-term potentiation, but they may also contribute to neuropathology by increasing glutamate excitotoxicity, inducing neuronal cell death, and oxidizing endogenous cannabinoids [15,16].

The most widely used graphical technique for assessing a biomarker’s diagnostic usefulness is the receiver operating characteristic (ROC) curve. The area under the curve (AUC) in ROC curves is a useful tool for summarizing the test’s overall diagnostic accuracy. AUCs are a useful metric that assesses a biomarker’s typical validity by combining measures of sensitivity and specificity [17].

Understanding the mechanism of the interaction of anti-mitochondrial antibodies (AMA-M2), anti-histone autoantibodies, and PLA2/COX-2 might assist to emphasize their roles in the etiology of this condition.

## 2. Results and Discussion

Medical researchers recognize that relying solely on a single biomarker for diagnoses may not be accurate [18,19]. Multiple biomarker tests are increasingly being conducted on individuals and aggregated into a single score to improve diagnostic accuracy for clinicians. A diagnostic test’s accuracy is often measured by its sensitivity and specificity, or the likelihood of true positives and false negatives for a certain threshold. The ROC curve, defined as sensitivity versus 100-specificity over all potential cut-points for a given biomarker [20,21], is a comprehensive figure that depicts the influence of a biomarker as the cut-point changes. Although clinicians without statistical training do not need to understand both the advanced mathematical equation and the analytic process of ROC curves, they must understand the core concepts of a ROC curve analysis in order to correctly use and interpret the diagnostic performance of either the independent or the combined variables. In general, an AUC of 0.5–0.6 suggests that a biomarker has no diagnostic or discrimination value, 0.7 to 0.8 is acceptable, 0.8 to 0.9 is excellent, and more than 0.9 indicates exceptional ability to diagnose patients with and without the disease or condition based on the ROC analyzed biomarker [17].

**Figure 1:**
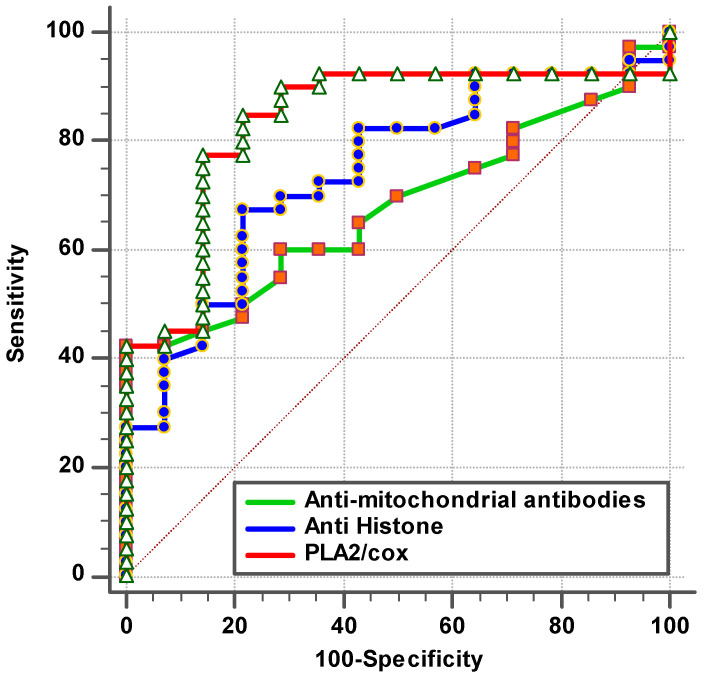
ROC curve for tested parameters of patient group according to control group.

**Figure 2:**
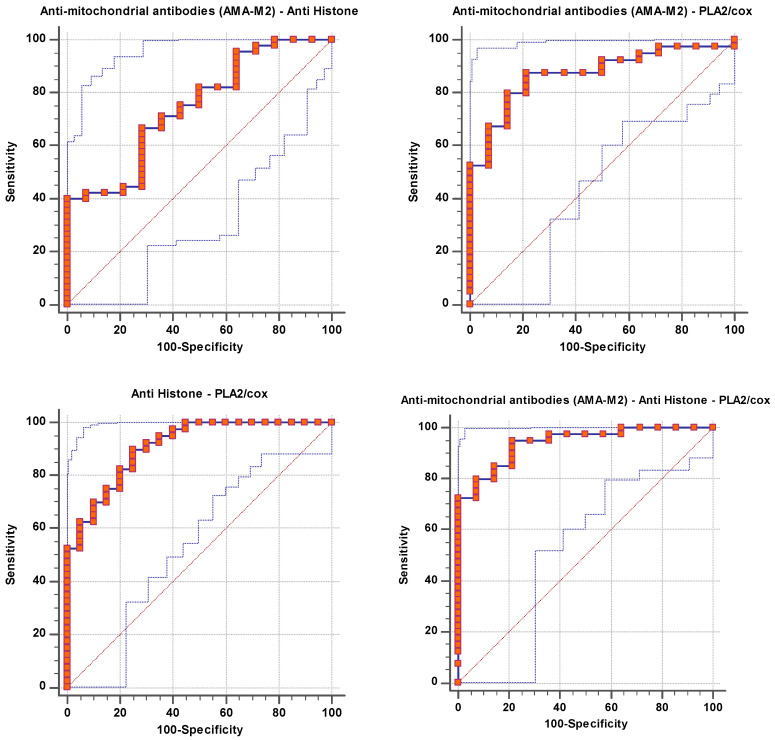
Combined ROC for tested parameters of patient group according to control group.

The remarkable increase in the independent AUC for AMA-M2 and PLA2/COX-2 from 0.663 and 0.869, respectively (Table 1 and Figure 1), to 0.871 when combining (Table 2 and Figure 2) could easily demonstrate the integrative contribution of both variables as biomarkers of mitochondrial dysfunction, impaired lipid metabolism, and neuroinflammation as etiological mechanisms of ASD.

**Figure 3:**
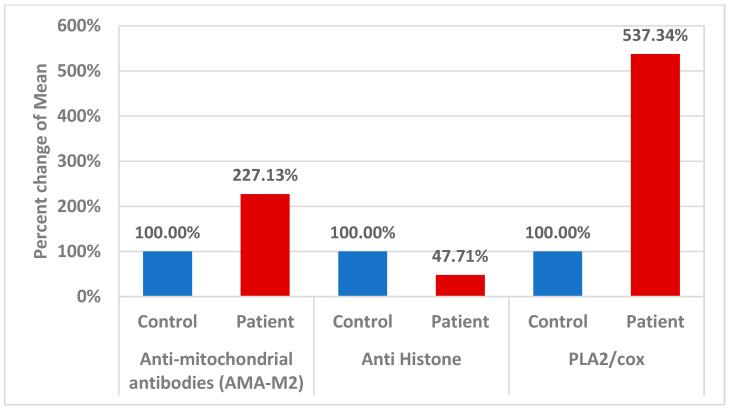
Percent change in mean for different parameters in control and patient groups.

According to fatty acid quantification data, autistic children exhibited high plasma levels of omega-6 AA but low levels of omega-3 eicosapentaenoic acid (EPA) and docosahexaenoic acid (DHA), which is reliable with evidence of aberrant lipid metabolism in neuropsychiatric diseases [22]. It has been suggested that this imbalance is caused by changes in the structure and function of phospholipids in cell membranes. Omega-3 fatty acids have been linked to the formation of eicosanoids, which have anti-inflammatory, antithrombotic, and vasodilator properties, and were lower in autistic children [23]. In contrast, biologically active pro-inflammatory compounds called prostaglandins are mostly produced from AA and are released from the membrane via the PLA2 enzyme; they appear to be especially linked to the physiological dysfunctions that are typical of ASD [24,25]. In fact, research suggests that the instability in fatty acid levels may be driven by an increase in PLA2 activity, possibly in conjunction with the elevated oxidative stress present in these patients [26,27]. In relation to neuroinflammation, variations in the COX-2 and PLA2 genes may enhance the risk of IFN-α-induced neurological illness via altering EPA and DHA levels [28]. The pathophysiology of autism is linked to mitochondrial malfunction, oxidative stress, and neuroinflammation [29,30]. In relation to the increased AMA-M2 reported in the current study (Table 3 and Figure 3), mitochondrial dysfunction is caused in part by proton gradient dissipation, which causes an increase in reactive oxygen species (ROS), uncoupling of the electron transport chain, decreased oxidative phosphorylation, decreased mitochondrial biogenesis, and altered mitochondrial dynamics [31].

In an attempt to explain the remarkable increase in AUC of combining AMA-M2 and PLA2/COX, and the positive correlation recorded between the two variables (Table 4), we can suggest that an inflammatory response is triggered by the suppression of mitochondrial respiratory chain complexes III and V in autistic individuals [32,33,34], which may be particularly significant in relation to the generation of prostaglandin E2 as a product of activated COX-2 and higher PLA2/COX-2 through mitochondrial Ca^2+^ exchange, the generation of ROS, and the activation of the nuclear factor kappa-light-chain-enhancer of activated B cells. These conclusions could be helpful in improving our understanding of how mitochondria function in the pathophysiology of ASD.

Table 3 describes comparison between the control group and patient group for each parameter using the Mann–Whitney Test (nonparametric data).

Massive amounts of histones are present in nucleated cells. Thus, even a small portion of the overall amount of histones released from the nucleus has the potential to affect how cells function. Interestingly, Cascone et al. [35] demonstrate that histones bind effectively to mitochondria, and destabilize the mitochondrial membranes, a mechanism that may convey genotoxic signals to mitochondria and promote neuronal apoptosis as another etiological mechanism of ASD following DNA damage. Based on this, the remarkable decrease in anti-histone autoantibodies recorded in the present study (Table 3 and Figure 3) might explain the increase in the AUC of combining AMA-M2 as a marker of mitochondrial dysfunction and anti-histone autoantibodies as a marker of the nuclear–mitochondria signaling pathway.

The documented significant negative correlation between anti-histone antibodies as an autoimmune marker and PLA2/COX as a marker of neuroinflammation (Table 4), as well as the notable rise in ROC-AUC of combining both variables (AUC = 0.909) (Table 2 and Figure 2) compared to AUCs of 0.77 and 0.869 for independent anti-histone antibodies and PLA2/COX-2, respectively (Table 1 and Figure 1), could help to suggest the important role of phospholipid-degrading enzymes’ inflammatory signaling in inducing autoimmunity. This may imply that immunological dysfunction and neuroinflammation are both likely to be important factors in the pathophysiology and etiology of ASD. The likelihood of having a child with ASD is greatly increased by maternal autoantibodies and inflammation during pregnancy, as well as by familial autoimmunity, which is a prevalent risk factor [36].

Table 2 and Figure 2 showed a significant increase in the AUC of combined ROC for AMA-M2/anti-mitochondrial antibodies/PLA2/COX. It yielded an outstanding AUC value of 0.941, indicating that the three tested biomarkers had a strong predictive and diagnostic value as a panel. In an attempt to explain the interaction between autoimmunity, mitochondrial dysfunction, and neuroinflammation as three signaling pathways related to the three measured variables, it was interesting to consider the effect of lipid metabolism.

Notably, there is a substantial correlation between elevated ASD and respiratory distress as well as other hypoxic markers during the prenatal and postnatal periods [37,38]. Under hypoxic conditions, a succession of events, including as excitatory transmitter release, extracellular Ca^2+^ influx, mitochondrial dysfunction, energy failure, and neuronal death, are commonly occurring. In the central nervous system, PLA2 is known to be overexpressed in neurons under hypoxia as a clinical phenomenon in autistic individuals [39]. Phospholipases are thought to have functions in brain development since they are intracellularly expressed in brain cells [40]. In an attempt to find the correlation between the high significant increase in PLA2/COX and the significant increase in AMA-2 as a marker of mitochondrial dysfunction, it is interesting to highlight the fact that PLA2 enzymes are particularly effective at hydrolyzing mitochondrial membranes, releasing AA, lyso-cardiolipin, and encapsulated mitochondrial DNA, which can induce neuroinflammation and neuronal cell death. Moreover, research conducted on animals revealed that catalytically active PLA2 was located on the inner face of the inner mitochondrial membrane. It was postulated that during hypoxia, elevated PLA2 activity resulted in a faster catabolism of mitochondrial phospholipids, which led to the membrane losing Ca^2+^ homeostasis, undergoing a permeability transition, and releasing cytochrome c into the mitochondria, which intensified the apoptotic pathway of neurons [39].

Table 5 presents the results of fitting the multivariate model including a test of significance for each predictor while controlling for all other variables already in the model.

It proved that the effect of PLA2/COX-2 and anti-histone as predictors of autism using standardized odds ratios (ORs) in the multivariate model differs in order and magnitude from that in the univariate model.

Table 5 describes the logistic regression test for the patient group as a dependent variable, with anti-mitochondrial antibodies (AMA-M2), anti-histone antibodies, and PLA2/COX-2 as independent variables, using the Enter method.

**Figure 4:**
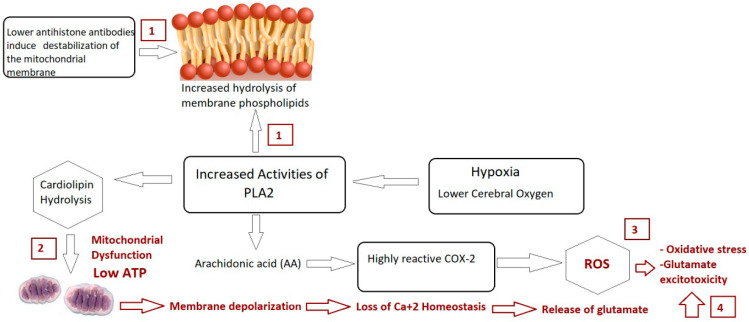
Illustrated mechanism of integrative role of PLA2/COX-2 and anti-histone autoantibodies in relation to mitochondrial dysfunction, oxidative stress, and glutamate excitotoxicity as etiological mechanisms of autism.

In conclusion, the significant rise in the predictive diagnostic value of combining three ASD biomarkers, AMA-M2, PLA2/COX, and anti-histone antibodies, along with their proven roles in mitochondrial dysfunction, autoimmunity, and glutamate excitotoxicity as the disorder’s three etiological mechanisms (illustrated in Figure 4) raise the possibility that the measured variables could be helpful diagnostic markers for ASD that could aid in early intervention.

## 3. Materials and Methods

The study protocol was authorized by King Saud University’s medical college ethics committee in accordance with the most recent Helsinki Declaration (Edinburgh, 2000). This study included two sets of participants: forty patients with ASD and forty age- and gender-matched healthy controls. The time frame for inclusion was May 2019 to December 2019. All participants provided informed consent, which was signed by the participants’ parents. Recruitment for both groups took place via the ART Center (Autism Research & Therapy Center) facility. All research participants had their ASD diagnoses confirmed using the Autism Diagnostic Interview-Revised (ADI-R), the Autism Diagnostic Observation Schedule (ADOS), and the 3DI (Developmental, Dimensional Diagnostic Interview).

This study included male children with autism who were 5.5 ± 2.2 years old. Each was a simplex case, meaning just one member of the family was afflicted. Everybody’s tests were negative for the fragile X syndrome gene. They have well-defined autistic phenotypes, with moderate-to-severe recorded values on the Social Responsiveness Scale (SRS) (70–76), mild-to-severe recorded values on the Childhood Autism Rating Scale (CARS), and a well-defined Short Sensory Profile. Forty control subjects, aged 5.3 ± 2.3 years, were selected from the Pediatric Clinic at the same institution based on their age and gender. Individuals with severe neurological abnormalities, fragile X syndrome, or any other medical issues precluded from this study were not allowed to participate. Parental interviews were used to assess all participants for past or current medical illnesses. While not following a specific gluten-restricted diet, all of the study’s patients and controls followed comparable but distinct diets.

### 3.1. Blood Sampling

After an overnight fast, a trained technician took blood samples into 3 mL blood collection tubes containing EDTA from children with ASD and healthy controls. Blood was centrifuged at 4 °C and 3000× *g* for 20 min right after collection. To prevent multiple freeze–thaw cycles, the plasma was divided into three 0.5 mL aliquots and kept at −80 °C until needed.

### 3.2. Ethics Approval and Consent 

This work was authorized by the ethics committee at King Khalid Hospital, King Saud University (approval number: 11/2890/IRB) on 13/01/2019. All study participants’ parents provided written consent in accordance with the ethics committee requirements.

### 3.3. Biochemical Assays

The most widely used ELISA technique involves introducing an aliquot of a sample or calibrator containing the antigen (Ag) to be tested and allowing it to bind with a solid-phase antibody (Ab). After washing, an enzyme-labeled antibody is added to form a solid-phase Ab-Ag-Ab enzyme “sandwich complex”. An enzyme substrate is administered after the unbound antibody has been removed with washing. The amount of product produced is closely correlated with the amount of the antigen in the sample.

#### 3.3.1. Anti-Histone Antibodies Assay

A Genway Biotech, San Diego, USA product, the ELISA kit, was used to quantify the anti-histone autoantibody. The samples were run concurrently with the controls and standards on the same run. If there were any highly purified human anti-histone-specific antibodies in the plasma, they would bind in the wells after 30 min of incubation at 25 °C. The wells were cleaned to get rid of the loose plasma antigens. Anti-human IgG conjugated with the enzyme horseradish peroxidase interacts with the attached patient antibodies immunologically, generating a conjugate/antibody/antigen complex.

#### 3.3.2. Plasma AMA-M2 Assay

The levels of plasma AMA-M2 in autistic patients and healthy control subjects were determined by an enzyme-linked immunosorbent assay (ELISA) (Euroimmun, LübeckGermany) according to the manufacturer’s instructions.

#### 3.3.3. Assay of cPLA2

The concentration of cPLA2 was determined using a competitive enzyme immunoassay approach developed by Amsbio and Blue Gene Company, Cambridge, MA, USA following the manufacturer’s instructions. The product’s detection range was 1.56 ng/mL to 100 ng/mL.

#### 3.3.4. Assay of COX-2

COX-2 levels were measured with a quantitative sandwich enzyme-linked immunosorbent assay (ELISA) kit from CUSABIO (8400 Baltimore Avenue, Room 332, College Park, MD 20740, USA). The measurement was carried out in accordance with the manufacturer’s specifications, and the minimum detectable dose was 0.31 ng/mL.

### 3.4. Statistical Analyses

This study analyzed data using IBM SPSS software version 22.0 (IBM Inc., Armonk, NY, USA). The Shapiro–Wilk Test was performed to ensure data normality for each group. Results were provided as the Minimum, Maximum, and Median. The Mann–Whitney Test was used to compare two nonparametric groups, with *p* ≤ 0.05 indicating a significant difference. The Spearman rank correlation coefficient (R) was used to correlate multiple nonparametric variables. ORs from the logistic regression analysis describe the relationship between biomarkers and the clinical state in the combined ROC curve. ROC curves were generated for each logistic regression model. The area under the curve (ROCAUC) was calculated for each marker and marker combination using a nonparametric technique.

## 4. Conclusions

Overall, this study has shown that it is possible to construct an appropriate model for the diagnosis of ASD in the future using quantitative predictors. The capacity to create a model for predicting ASD would have a substantial impact on existing diagnostic processes, and this study provides a preliminary look at how our blood may provide the clues required to solve the problem.

## Figures and Tables

**Table 1:** ROC results for patient group according to control group as a reference group.

Parameters	AUC	Cut-Off Value	Sensitivity %	Specificity %	*p* Value	95% CI
Anti-mitochondrial antibodies (AMA-M2)	0.663	0.162	40.7%	100.0%	0.062	0.530–0.796
Anti-histone antibodies	0.770	0.672	71.1%	75.0%	0.001	0.653–0.887
PLA2/COX	0.869	0.205	85.0%	85.0%	0.001	0.780–0.959

**Table 2:** Combined ROC results of the anti-mitochondrial antibodies (AMA-M2), anti-histone autoantibodies, and PLA2/COX for patient group according to control group as a reference group.

Parameters	AUC	Sensitivity %	Specificity %	*p* Value	95% CI
AMA-M2 with anti-histone antibodies	0.743	40.0%	100.0%	0.006	0.601–0.885
AMA-M2 with PLA2/COX	0.871	87.5%	78.6%	0.000	0.774–0.969
Anti-histone antibodies with PLA2/COX	0.909	90.0%	75.0%	0.000	0.834–0.984
AMA-M2 with anti-histone antibodies with PLA2/COX	0.941	95.0%	78.6%	0.000	0.881–1.000

**Table 3:** Comparison between control group and patient group for each parameter.

Parameters	Groups	N	Min.	Max.	Mean ± S.D.	Median	Percent Change	*p* Value
Anti-mitochondrial antibodies (AMA-M2)	Control	14	0.08	0.11	0.10 ± 0.01	0.10	100.00	0.050
	Patient	54	0.08	0.33	0.22 ± 0.03	0.10	227.13	
Anti-histone antibodies	Control	20	0.25	7.70	1.82 ± 1.84	1.15	100.00	0.001
	Patient	45	0.05	8.13	0.87 ± 1.47	0.37	47.71	
PLA2/COX-2	Control	40	0.00	0.56	0.11 ± 0.13	0.07	100.00	0.001
	Patient	40	0.00	3.07	0.62 ± 0.58	0.44	537.34	

**Table 4:** Correlations between the measured parameters using Spearman Correlation.

Parameters	R (Correlation Coefficient)	*p* Value	
Anti-mitochondrial antibodies (AMA-M2) with anti-histone	0.043	0.746	P ^a^
Anti-mitochondrial antibodies (AMA-M2) with PLA2/COX-2	0.087	0.533	P ^a^
Anti-histone with PLA2/COX-2	−0.013	0.921	N ^b^

^a^ Positive Correlation; ^b^ Negative Correlation.

**Table 5:** Logistic Regression of autistic individuals’ group.

Parameters	Regression Coefficient	Standard Error	Odds Ratio	95% CI for Odds Ratio		*p* Value
				Lower	Upper	
Anti-mitochondrial antibodies (AMA-M2)	55.540	25.335	1.32 ×10^24^	359.336	4.85 × 10^45^	0.028
Anti-histone	−0.501	0.237	0.606	0.381	0.965	0.035
Anti-mitochondrial antibodies (AMA-M2)	63.070	34.255	2.46 × 10^27^	0.017	3.54 × 10^56^	0.066
PLA2/COX-2	5.823	2.163	337.866	4.868	23,449.68	0.007
Anti-histone	−1.090	0.378	0.336	0.160	0.706	0.004
PLA2/COX-2	8.819	2.653	6.76 × 10^3^	37.325	1.22 × 10^6^	0.001
Anti-mitochondrial antibodies (AMA-M2)	76.848	39.487	2.37 × 10^33^	0.580	9.69 × 10^66^	0.052
Anti-histone antibodies	−1.624	0.583	0.197	0.063	0.618	0.005
PLA2/COX-2	10.503	3.702	3.64 × 10^4^	25.726	5.15 × 10^7^	0.005

## Data Availability

The original contributions presented in the study are included in the article, further inquiries can be directed to the corresponding author.

## Limitations

A disadvantage of the current study is the limited size and changes in the number of evaluated samples caused by insufficient samples.
